# Process–Structure–Property Relationship Development in Large-Format Additive Manufacturing: Fiber Alignment and Ultimate Tensile Strength

**DOI:** 10.3390/ma17071526

**Published:** 2024-03-27

**Authors:** Lucinda K. Slattery, Zackery B. McClelland, Samuel T. Hess

**Affiliations:** 1 Engineer Research and Development Center, U.S. Army Corps of Engineers, 3909 Halls Ferry Road, Vicksburg, MS 39180, USA; zackery.b.mcclelland@erdc.dren.mil; 2 Department of Physics and Astronomy, University of Maine, 5709 Bennett Hall, Orono, ME 04469, USA; samuel.hess@maine.edu

**Keywords:** additive manufacturing, material extrusion, X-ray microscopy, microstructure, fiber alignment, thermoplastic, tensile strength

## Abstract

Parts made through additive manufacturing (AM) often exhibit mechanical anisotropy due to the time-based deposition of material and processing parameters. In polymer material extrusion (MEX), printed parts have weak points at layer interfaces, perpendicular to the direction of deposition. Poly(lactic acid) with chopped carbon fiber was printed on a large-format pellet printer at various extrusion rates with the same tool pathing to measure the fiber alignment with deposition via two methods and relate it to the ultimate tensile strength (UTS). Within a singular printed bead, an X-ray microscopy (XRM) scan was conducted to produce a reconstruction of the internal microstructure and 3D object data on the length and orientation of fibers. From the scan, discrete images were used in an image analysis technique to determine the fiber alignment to deposition without 3D object data on each fiber’s size. Both the object method and the discrete image method showed a negative relationship between the extrusion rate and fiber alignment, with −34.64% and −53.43% alignment per extrusion multiplier, respectively, as the slopes of the linear regression. Tensile testing was conducted to determine the correlation between the fiber alignment and UTS. For all extrusion rates tested, as the extrusion multiplier increased, the percent difference in the UTS decreased, to a minimum of 8.12 ± 14.40%. The use of image analysis for the determination of the fiber alignment provides a possible method for relating the microstructure to the meso-property of AM parts, and the relationship between the microstructure and the properties establishes process–structure–property relationships for large-format AM.

## 1. Introduction

Additive manufacturing (AM) is one of the fastest-growing manufacturing methods because of the geometric customization opportunities, strength of printed polymers, and high manufacturing speed of printing [1]. Large-format additive manufacturing (LFAM), which is defined by a build volume of 1 m^3^ or greater [2], has been used to rapidly produce large-scale parts in various industries, such as aerospace, construction, and architecture [3]. Thermoplastic LFAM printers are often gantry-based and pellet-fed to allow for a high throughput, but this can lead to a decreased geometric resolution [4] and complex thermal mechanical behavior. As shown in [5], the relationship between the bead volume and degree of crystallinity in a semi-crystalline thermoplastic polymer as a result of processing temperatures and time within the extruder resulted in a variable mechanical performance. Previous work has found printing and processing parameters to have an impact on the component’s mechanical and thermal performance [6,7,8,9]. This shows a process to performance dependence, which is often fully defined as the process–structure–property relationship [10]. The process–structure–property relationship in materials is not AM-specific and was outlined for steel in Olson et al. as the dependence of performance on the material’s properties, which are a function of structures induced during processing [10,11]. To produce parts with a predictable performance, it is fundamental to understand process–structure–property relationships, but these relationships are still under-defined in thermoplastic AM [12] and are being actively studied [9,13,14,15,16]. However, the authors are unaware of any process–structure–property relationship development research in thermoplastic LFAM. Previous work by Owens et al. (2022) shows a significant difference in temperatures, dominant heat transfer terms, and other processing conditions for LFAM compared to small-scale AM, highlighting the need for LFAM-specific process–structure–property mapping [17]. Owens et al. developed a reduced dimension numerical model to simulate the thermal behavior of fused filaments in small-scale AM and in LFAM. The findings showed that when modeling LFAM, unlike in small-scale AM, there is a non-negligible radiative heat transfer term and large temperature differentials within a bead, where the surface cools rapidly but the center of the bead stays hot. This work highlighted key differences in the material response to processing at the different length scales associated with LFAM and small-scale AM. As a result, developed process dependencies cannot be translated from small-scale AM to LFAM. Furthermore, Robles Poblete et al. found that in LFAM single bead walls, to match FE-predicted temperatures with experimentally measured temperatures with intra-bead thermocouples, the FE model required a variable convection coefficient [18]. The same was not found for the small scale, where Sinha et al. used a single convective coefficient and a finite difference model to predict intra-bead temperature values and validated the model with embedded thermocouples [19]. The variation in modeling techniques required from small-scale AM to LFAM shows a possible process change between the two technologies and shows that process–structure–property relationships developed for small scales cannot be translated to LFAM without experimentation.

**Figure 1:**
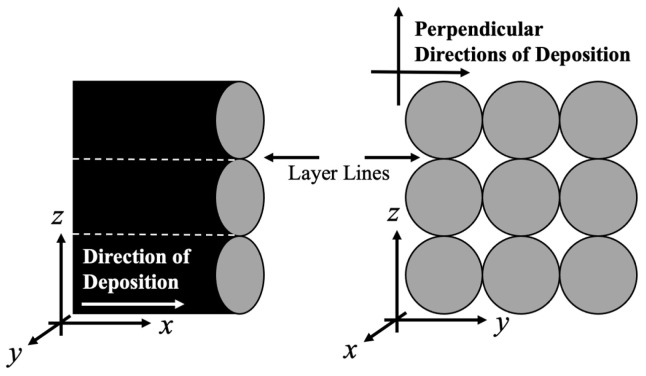
Visualization of layer lines and direction of deposition in additively manufactured part; typically, the direction of deposition is the strongest orientation whereas the perpendicular directions of deposition are the weaker orientations (with z being the weakest) from the time-based deposition process and dissimilar material (i.e., mesoscale voids) in the testing direction.

A common reason for mechanical failure in polymer additive manufacturing is poor layer adhesion [19,20,21,22,23]. The weakness at layer interfaces is caused by the requirement of both the previously deposited layer and the newly deposited layer being above the glass transition temperature (T_g_), as well as the previously deposited bead being at a low enough temperature to maintain dimensional stability as the new layer is deposited. This leads to limited adhesion between subsequent layers because of the short time duration where both bonding and dimensional stability can occur. As pictured in Figure 1, the layer lines are planes of material inhomogeneity from the time-based deposition process. Current research suggests that, depending on processing conditions, fibers align highly in the direction of deposition as opposed to perpendicular to the direction of deposition, and, as a result, the tensile strength in the direction of deposition increases [24,25,26,27,28,29,30,31,32,33,34,35].

The addition of short fibers to a polymer matrix in additive manufacturing can increase the mechanical performance of the resultant parts without modifications to current extrusion-based printers [36]. Unlike short fiber thermoplastics (SFTs), a continuous fiber placement in additive manufacturing often requires modifications to standard MEX (material extrusion) equipment to lay the fibers [37]. Due to the discontinuous nature of SFs (short fibers), the fibers orient as a function of the flow field through the polymer matrix, which can impact the mechanical performance and deformations [6,38].

**Figure 2:**
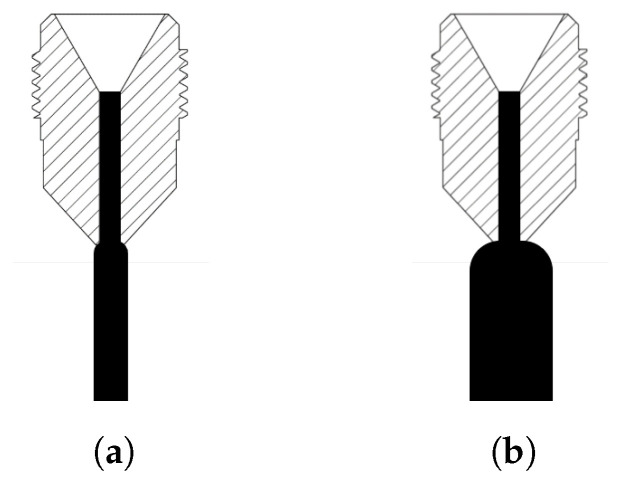
Characterization of extrusion rates relative to nozzle geometry, where the nozzle pictured is accurate to the internal structure of the nozzle used in this work: (**a**) low extrusion multiplier (EM less than 1.0); (**b**) high extrusion multiplier (EM greater than 1.0).

In polymer MEX, for a given nozzle geometry and print setting, a high extrusion rate creates a larger bead width relative to a low extrusion rate, as shown in the difference in extrusion width in Figure 2. It has been shown that a larger bead width provides stronger layer interfaces [7] and therefore impacts the ultimate tensile strength [39], due to many factors, such as an increased contact area between layers and an increased thermal mass to allow for a longer bonding time where both layers are above T_g_ [21,40]. Given the decrease in pressure at the nozzle die, this work hypothesized that a larger extruded bead relative to a given nozzle generates a larger force on the fibers in the polymer matrix as expansion occurs, as seen in [41] for cementitious materials where the impact of the flow rate on the fiber alignment was modeled. It has been found in filament small-scale AM that the extrusion width was the primary factor impacting the fiber alignment of the deposition [42], but LFAM is still to be explored. The purpose of this research is to evaluate the influence of the extrusion multiplier (EM) on the fiber alignment in LFAM MEX experimentally, via two methods, and relate it to the ultimate tensile strength of printed parts to produce a set of process–structure–property relationships.

## 2. Materials and Methods

**Figure 3:**
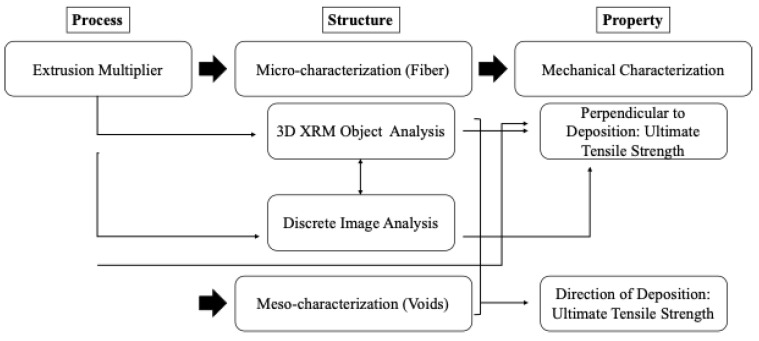
Process structure property map for experimentation.

The process was varied through the modification of the EM, and the resultant structure was measured both at the microstructure level through fiber alignment measurements and the meso-structure level through density corrections due to bead geometry. The ultimate tensile strength in two orientations relative to the deposition was the property measured as a result. The process–structure–property experimentation mapping is presented in Figure 3, following the process, structure, and property definitions in Olson et al. [11].

### 2.1. Coordinate System

The experimentation focused on a 2-coordinate analysis for the UTS, the direction of deposition and perpendicular to the direction of deposition. The present work refers to the *x* orientation in Figure 1 as the direction of deposition and the *z* direction as perpendicular to the direction of deposition for the UTS. The fiber alignment was reported as a percent value of the orientation in the direction of deposition, *x*.

### 2.2. Print and Printer Specifications—Process

**Figure 4:**
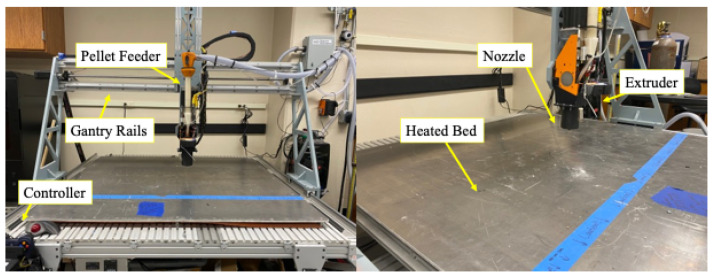
Large-format pellet printer used for all experimentation with MDPH2 extruder from Massive Dimensions, Barre, VT USA, a heated bed, and 3 mm diameter nozzle.

The material studied was pelletized poly(lactic acid) with a 10% short carbon fiber additive (4043D PLA with a 10% CF additive). The same printer was used to print all specimens and is pictured in Figure 4. The printer was built by engineers at the Engineer Research and Development Center, U.S. Army Corps of Engineers, Vicksburg, MS USA, and is used for research in LFAM.

**Figure 5:**
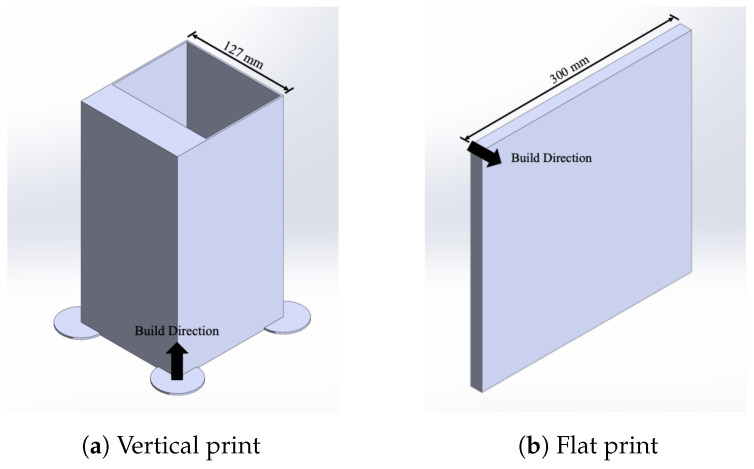
Print geometries: (**a**) produced tensile samples perpendicular to the direction of printing; (**b**) produced tensile samples in the direction of printing that lie flat on the bed.

The prints were all sliced with a layer height of 2 mm with a print speed (feed rate) of 650 $\frac{\mathrm{mm}}{\min}$. The nozzle temperature (T_melt_) was set to 230 °C with a bed temperature of 60 °C. Two geometries were chosen to meet the required volume and to capture the multiple orientations of tensile samples desired with minimal wasted material. Both print geometries are shown in Figure 5. All prints were sliced in a PrusaSlicer, developed by Prusa Research, Prague, Czach Republic, version 2.3.3 with tool path allotted bead widths of 2.20 mm and 3.38 mm for the perimeter and infill, respectively. The perimeter was used to help with the infill geometric fidelity of the desired geometry and adhesion to the bed but was not a feature in any samples tested. All infill speed/feed rates were constant for every print.

The value of the extrusion rate is very specific to the extruder being used, whereas the extrusion multiplier (EM) is a unitless ratio of the expected bead width and tool path-informed bead width [43]. For example, with an EM of 1.1, the expected bead width is 1.1 times larger than the tool path allotted for the given bead. While each printer–extruder–material system may require a unique EM for geometric fidelity, an EM of 1.0 would typically be used for a model-accurate print because the desired bead width would equal the expected bead width. For relatively low extrusion rates, the resultant bead geometry is expected to have a bead–nozzle area ratio that is low for a given printer. Comparatively, for relatively high extrusion rates, the resultant bead area is often larger than the area of the nozzle. This is visualized in Figure 2.

It is assumed the two geometries produced similar fiber alignment and tensile properties because the printing parameters were the same, excluding the geometry. While this assumption does not correct for the variation in the layer time between the two prints leading to varying interlayer adhesion, comparisons are not drawn on a geometric basis but rather for trend behavior for the same print with varying EMs. It is assumed that for these same geometries, if the tool path were varied, the tensile strength would also vary. As shown in Kim et al., the mechanical performance of parts could be tailored by the adjustment of the tool path for varying boundary conditions in short fiber AM  [44]. This is possible due to the anisotropic nature of additive manufacturing and the induced anisotropy from processing conditions on fibers in the polymer matrix.

**Figure 6:**
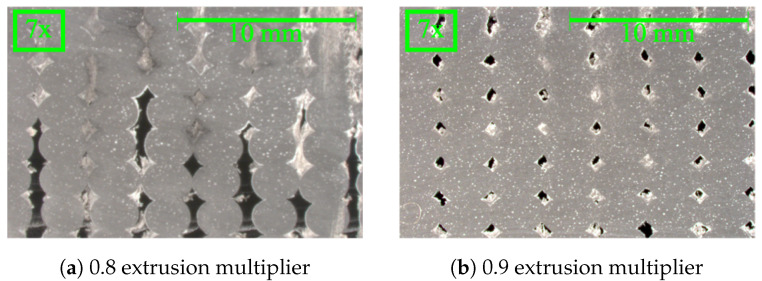

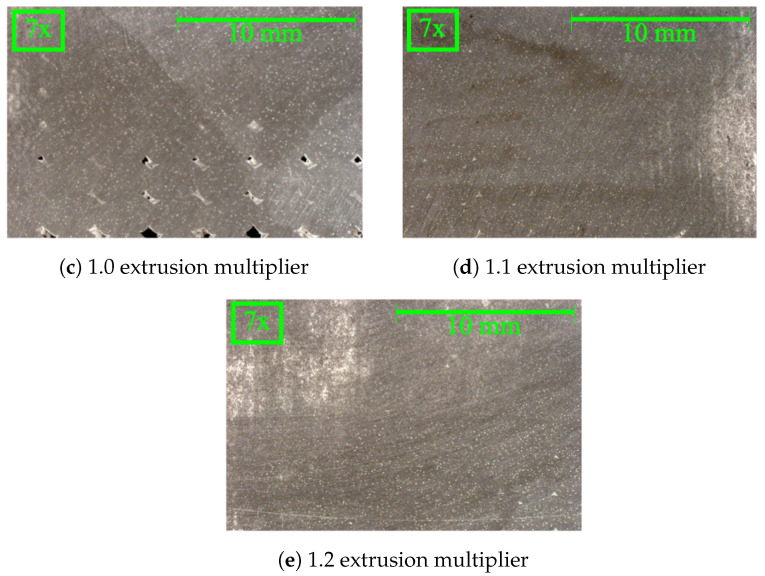
Sample prints of extrusion multipliers chosen for experimentation, polished for imaging.

In MEX, if a larger bead is desired for a part, the slicer settings are usually adjusted to compensate. However, slicers often use interrelationships between input parameters and the resultant extrusion and tool path behavior, so adjusting settings could have an unexpected impact on various other printing behaviors. As a result, only the EM was varied between prints, given it does not impact the tool path. The EMs chosen for the prints varied from discrete and visible discernment beads (Figure 6a) to fully fused materials (Figure 6e). Initially, solid panels were printed to determine viable EM ranges. Figure 6 shows the EMs that were viable for printing, as determined by the machining and polishing process. The samples were printed with the same infill settings as the prints later completed for tensile testing and used to determine desirable print settings. These samples were cut with a circular saw and polished from 800 to 1200 grit polishing paper on a Struers LaboForce-100 polisher. As seen in Figure 6a,c, the void space between each bead changes as a function of the location in the image. This is caused by both the cutting process unevenly distributing polymer dust into the void spaces, which is easily seen in Figure 6a, and the uneven compression of layers close to the build plate caused by the gravity of later layers in the deposition process. It is noteworthy that an EM of 0.7 was deemed not viable, as it fractured during the machining process.

### 2.3. Fiber Alignment Microstructure

As shown in Figure 2, the extrusion multiplier for a given nozzle impacts the resultant material deposited. The average fiber alignment was hypothesized to change as a function of the extrusion rate due to the expansion at the die of the nozzle generating a force, causing the fibers to rotate out of alignment with the direction of deposition. The fiber alignment was measured using two methods: 3D XRM analysis and 2D discrete image analysis, which is frequently used with optical microscopy and polished samples. The initial physical measurement was taken in a Carl Zeiss X-ray Microscopy Inc., White Plains, NY USA Xradia Versa X-ray Microscope (XRM). As the sample rotated, voxels were assigned a value based on the measured density. After the physical measurement had been completed, ORS Dragonfly Pro was used for the training and application of the deep learning model for object segmentation and material classification. The 2D image analysis was conducted on five images taken from each EM scan and input to a MATLAB script that conducts shape and grayscale (equivalent to density) thresholding, infers the projection to 3D, and calculates the fiber alignment with a set direction. For both methods, the alignment of the fibers is reported in percentage of alignment with the direction of deposition.

**Figure 7:**
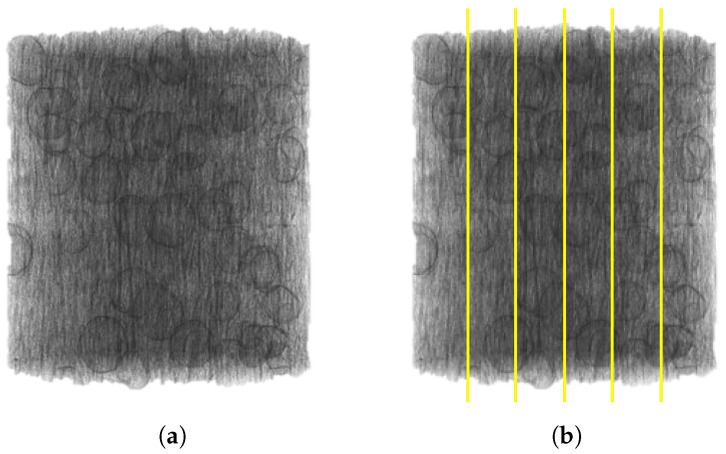
Difference between XRM scan and discrete image data: (**a**) XRM scan displaying voxels that correspond to fiber and pore; (**b**) approximate distribution of discrete images from XRM scan for image analysis, where the yellow shows this location.

The discrete images from an XRM scan were chosen, as shown in Figure 7, as opposed to optical microscopy on polished samples from the same print, to guarantee that the image analysis was performed on both equally distributed images and incorporated fibers that were captured in the XRM scan so the comparison between the two methods was not location-dependent relative to the print. Furthermore, during the cutting and polishing process, fibers could be removed or moved, which would lead to an inaccurate representation of the fiber alignment. This was performed in accordance with Sun et al.’s work, where 2D assumptions through discrete image analysis were applied to images from an XRM scan, as opposed to optical microscopy, for fiber orientation measurement in 30% fiber polypropylene injection-molded parts [45]. The MATLAB code was written for an XRM density-based image but could possibly be adjusted through the binary threshold value for an optical microscopy image with a high resolution between the fiber, polymer matrix, and pore. While this work compares XRM images to 3D reconstruction data, the assumptions made have been validated for optical microscopy techniques in Hanhan et al. [46]. The two fiber alignment techniques were compared using a Monte Carlo simulation to evaluate the validity of the assumptions required for 2D discrete image analysis. A simple Monte Carlo simulation (Appendix E) was run on both the image analysis and XRM reported fiber alignment values as a function of the EM to generate a distribution of possible slopes if each data point was randomly and normally located in the respective uncertainty bounds for a set number of permutations, with the method outlined in Raychaudhuri (2008)  [47]. The Monte Carlo simulation was run for various permutations to ensure that the permutation count did not drastically impact the resultant slopes. At each permutation count, the range of slope values was checked for overlap between the two distributions. It is important to note that the slope values were randomly generated, and, therefore, regenerating the same permutation count could slightly vary the resultant mean slopes.

#### 2.3.1. X-ray Microscopy

**Figure 8:**
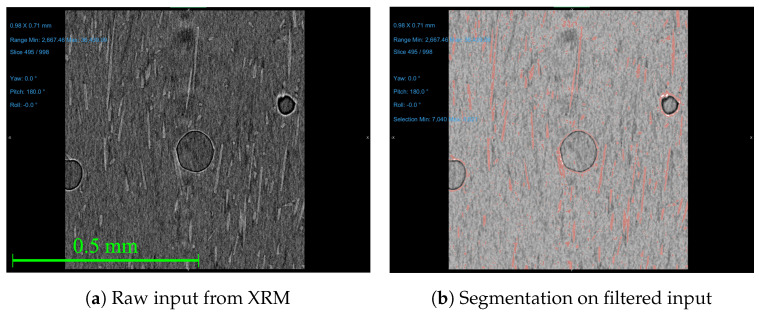
Various image filtrations and segmentation from XRM without deep learning. The red pixels correspond to predicted fiber pixels and the various shades of gray correspond to the density of the voxel relating to this cross-section. The red pixels surrounding the pores show that density-based sorting results in false recognition of those pixels as fibers. The images have the same scale.

The scan results were processed in ORS Dragonfly Pro, version 2022.2 from Comet Technologies, Montreal, Canada, a software used for XRM processing and analysis associated with Zeiss, White Plains, NY, USA. The scan was conducted with a 20X Objective lens through no source filter and set to an 80 kV voltage and 125 μA current. For the 0.8–1.0 EMs, there were 3201 projections, and for the 1.1 and 1.2 EMs, there were 4501 projections. The exposure time for all scans was set to 2.5 s. These scan settings were chosen to produce a good resolution with the sample composition and size. The scan data were post-processed with a MATLAB code to determine the Cartesian fiber orientation to provide an average fiber alignment in the direction of deposition for the scans. Because of the large scan and computational time, one specimen at each extrusion multiplier was scanned using an XRM to determine the fiber alignment. The specimen and bead scanned is assumed to be representational for the other samples, given the printing parameters are constant across one extrusion multiplier. To process the scans, the voxels were segmented into different object categories: polymer, pore, and fiber. This segmentation is usually performed by selecting voxels of a given density and assigning a material classification, but this process does not work if there is an overlap in density for material classifications in each scan. In polymer composites, the densities and resultant grayscale values for the voxels are not easily segmented and categorized, as shown in Figure 8.

As shown in Figure 8, the density-based segmentation cannot separate the fiber from the polymer and pore space. In the raw input data (Figure 8a) the segmentation creates a “flare” around the pore, as represented by the red highlight surrounding the circular pore in the center of the 2D scan image and the static noise of small red highlighted pixels throughout the entire sample in Figure 8b. This is due to the similarity in density for the polymer and the fiber. While the filtered image (filtered with the mean, median, and mean process with a kernel size of 5) does show less noise, there is still an overlap in the voxel grayscale values for the pores and fibers, requiring a more customized and sophisticated segmentation model. Due to the minimal difference in density of the polymer and fibers in additive manufacturing composites, density-based sorting, the primary segmentation method for XRM results, was not able to be used. To resolve this, a deep learning model was trained to segment the voxels into their respective material classifications. Similarly, due to some unanticipated optical behavior witnessed in the samples, the deep learning algorithm had to be individually trained for each sample. While there are other techniques to perform this advanced segmentation, like Artificial Intelligence and Machine Learning modules, such as ilastik, the authors were familiar with the deep learning module in Dragonfly ORS [48]. Future work should compare the various methods to segment these complex composites. The deep learning model was trained to perform density and shape-based segmentation to separate the fiber, polymer, and pore voxels. Each voxel was user-defined as a polymer, pore, or fiber for approximately 10 slices dispersed through the scan. The user definition was assisted by the Random Forest Morphological Gaussian MS Neighbors and Random Forest AM predictive models that were trained, applied, and manually corrected. The training was set to run for 100 epochs or until the loss-validation function was at the threshold value, as set by ORS Dragonfly Pro. After the model was trained, the results were reviewed manually to check for incorrect voxel identification by the model. In some cases, more training slices were generated, and the model was retrained until the slices that were randomly checked had correct segmentation. Within ORS Dragonfly Pro, the fiber ROI was then separated into individual objects based on their connected voxels; if an object was larger than 26 connected voxels (in ORS Dragonfly Pro), it was generated into an individual object. The fiber length distributions and volume compositions of each scan were used to evaluate the accuracy of the deep learning model and ensure consistency in segmentation across all EMs. Using the XRM processing script (Appendix D), the fiber alignment for each EM was reported. These values, like the 2D analysis, are impacted by user-defined inputs for thresholding. The thresholding used for the XRM processing is for both the volume of the objects and the voxel grayscale to correct for small noise that was incorrectly segmented as an ROI and large connected components that cannot be separated. Where more accurate data for connected components are desired, a water shedding technique is recommended.

#### 2.3.2. Image Analysis

**Figure 9:**
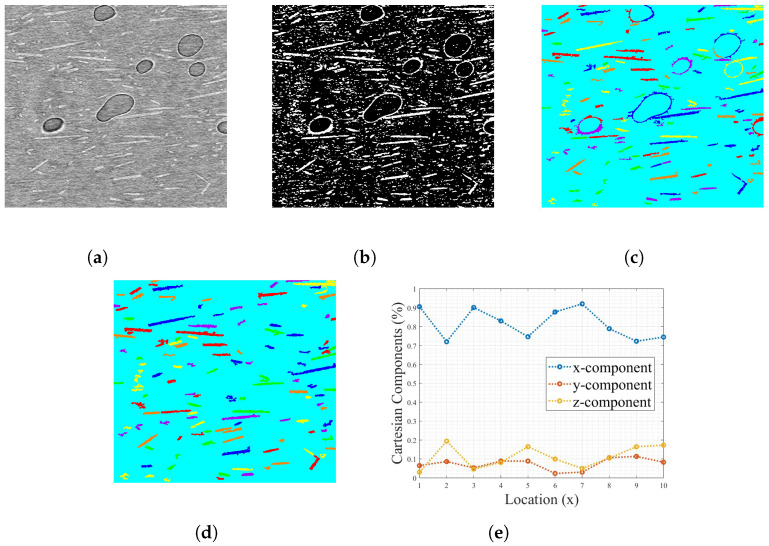
MATLAB image processing script, where the initial input was an image from XRM and the final output was the plot of fiber orientation relative to the image axis in Cartesian coordinates: (**a**) 2D plane from XRM; (**b**) binarized 2D Plane from XRM; (**c**) application of noise filter and object recognition, where the different colors show the recognition of unique objects; (**d**) application of shape filter; (**e**) resultant output of fiber orientation. A scale bar in this image processing is negligible, given that the maximum fiber length in a section is used to normalize all other visible fibers.

Images taken from the XRM scan were used in the 2D analysis MATLAB R2022b and R2023a script to determine the fiber orientation using a 2D projection-based analysis method. Five images from each scan were processed to determine the Cartesian orientations and calculate an average fiber alignment with the deposition, as shown in Figure 7. While optical microscopy and polishing techniques are most frequently utilized for the 2D method, as in Sharp et al. [49] and Fischer et al. [50], the XRM results in a 2D plane were used to ensure the same location was used to make the fiber measurements and that the fibers were not disturbed during the optical microscopy polishing process. The use of image analysis of a 2D plane for the determination of fiber orientations is used frequently and can assume a more complex shape of fibers than the code in this work. The MATLAB script image processing is visualized in Figure 9. In comparison with Sharp et al. [49] the 2D code written in this work is a simplistic approach with only two error-based tolerances: the shape and pixel values. Both the Sharp et al. and 2D code written for this assume fibers in a given image are all the same length, which is required for a 2D analysis; however, Sharp et al.  [49] treats the fibers as “kidney bean”-shaped in the cross-section as determined experimentally, but the code in this work uses a circular cross-section assumption, as used in Hanhan et al. [46]. The addition of complexity, such as a different cross-section shape, to the 2D image method could produce a higher accuracy in fiber recognition and alignment values. Benefits of the use of image analysis, as opposed to XRM segmentation and reconstruction, are accessibility, a vastly decreased computation time and decreased required computational resources. Despite these benefits, a 2D image analysis cannot correct for a bias in orientation for all fibers in an image away from the image plane or for a relationship between the fiber length and orientation where, for example, longer fibers may align with the direction of deposition more often than shorter fibers. The influence of a relationship between the fiber length and orientation is assumed to be negligible where there is a large count of fibers in an image plane and an average value is reported. Future work should explore the relationship between the fiber length and the alignment with the deposition, as well as this relationship’s impact on the accuracy of discrete image methods.

For the 2D code used, initially, an image is loaded into the MATLAB script and displayed for the user. The image is then binarized from a user-input thresholding value to determine where the designation in grayscale falls for the assignment of white and black pixels (zeros and ones in matrix form), as displayed in Figure 9b. The binary image is then passed through a noise filter that is user input-based to eliminate independent pixels with a connectivity lower than a thresholding value. The white pixels are sorted into individual objects based on connectivity. Following the noise filter, seen in Figure 9c, the objects are all measured for their area and compared against the theoretical value of an ellipse with the same semi-major and semi-minor axis as the objects. The percent difference is calculated, and, given an input error tolerance, shapes with a high percent difference are discarded. The shape-based segmentation is to eliminate the flare of pixels seen around pores in Figure 9c, and the elimination is shown in Figure 9d. Based on the user input, the image is sectioned into equal pixel widths. For all images, the section count was set to 10. The objects’ orientations are then calculated, using the assumption that all fibers in each section are the same length, and averaged to determine a section’s alignment vector, similarly to the orientation vector in Fischer et al.  [50]. The lengths of the fibers are determined by the longest fiber in the section; therefore, each section has a different assumed fiber length value. The maximum fiber length shown in the section is used as the normalizing length, assuming there are enough fibers in a section that the maximum can represent the average and produce an average value. This method cannot correct for all fibers in a section having the same alignment offset from the direction of deposition, because all fibers would have a bias shortening their apparent 2D length. Figure 9e shows the output to the user, where, based on an input value of sections, the script takes sections of equal size from the image and displays the average fiber orientation for this section. The orientation for each image section was then averaged for an image alignment. The five images were averaged to determine the orientation for each EM.

### 2.4. Density Image Analysis—Meso-Structure

**Figure 10:**
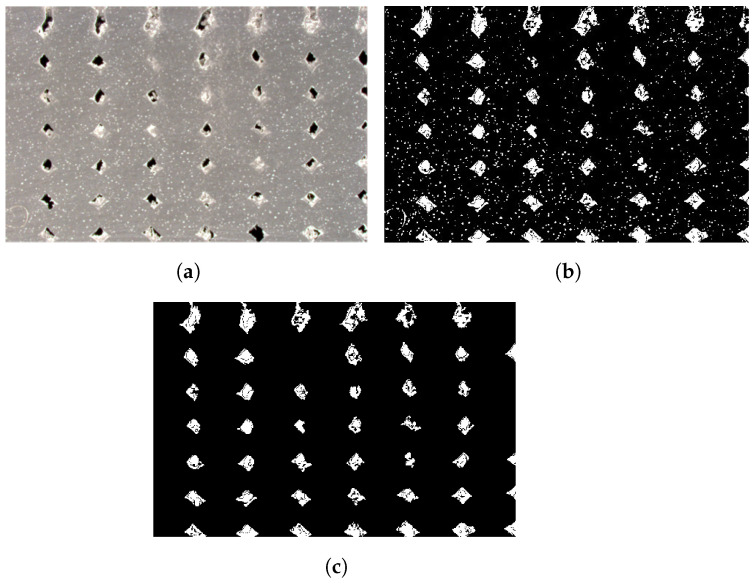
Sample image processing (0.9 EM) for area estimation for tensile strength normalization (the scale is negligible as the reported material content is a percentage value based on pixels identified as void to material): (**a**) initial image (0.9 EM)—cropped; (**b**) selection of extreme light and dark pixels in ImageJ for void recognition; (**c**) object recognition of voids from MATLAB processing script.

To produce an ultimate tensile strength that was representative of the microstructure within the bead (fiber alignment), as opposed to the meso-structure (voids), the load-bearing cross-sectional area was estimated for tensile samples in the direction of deposition. A sample of the area estimation process is shown in Figure 10. Assuming the images in Figure 6 are representative of the cross-sections of the direction of deposition tensile samples, an area ratio of material to total area was estimated. The images were minimally cropped to limit the effect of non-uniform voids due to compression towards the bed, as seen in the difference in material density in Figure 6c. The images were binarized (8-bit) and thresholded to capture the darkest pixels, which corresponded to an empty void, and the lightest pixels, which corresponded to a void filled with powdered material from the cutting process. The binary image was processed in MATLAB to set a minimum connected pixel value to disregard small intra-bead pores, as seen as the speckling in the beads in Figure 10a,b. The utilized MATLAB script is detailed in Appendix F, where the image has already been thresholded in ImageJ. The recognized void content for the 0.9 EM is shown in Figure 10c.

The resultant percentages are assumed to be an estimation—not an accurate and exact measurement of the cross-section material percentage—given the prints had different geometries and the results are dependent on the correct identification of pixels. The results of the ultimate tensile strength adjusted for the estimated material percentage in the cross-sectional area were reported, and a linear regression was fit to develop a microstructure to meso-normalized ultimate tensile strength relationship. The same adjustment cannot be applied to perpendicular to the direction of deposition because the cross-section is not reflective of the material percentage in a uniform way but rather varies as a function of the height in the sample. The high locational dependency in the samples is visualized in Figure 6a, where some portions of the image have much larger voids at layer lines than sections at the widest point in the bead.

### 2.5. Mechanical Testing—Property

**Figure 11:**
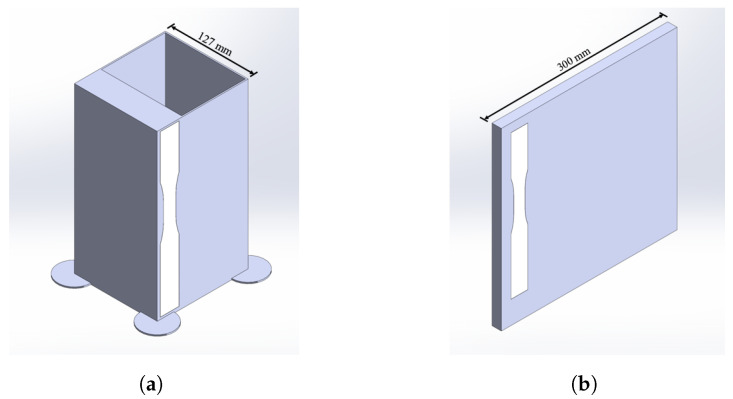
Print geometries showing the dogbone orientations relative to the prints: (**a**) produced tensile samples perpendicular to the direction of deposition; (**b**) produced tensile samples in the direction of deposition that lie flat on the bed.

**Figure A1:**
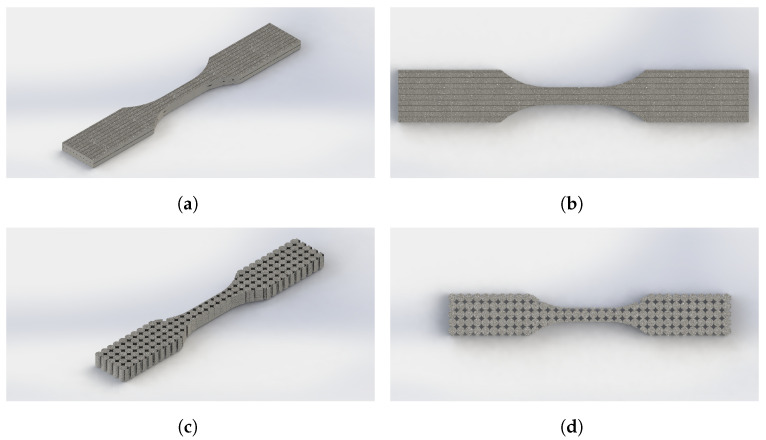
Dogbone specimens for tensile testing: (**a**) parallel to direction of deposition (isometric view); (**b**) parallel to direction of deposition (top view); (**c**) perpendicular to direction of deposition (isometric view); (**d**) perpendicular to the direction of deposition (top view).

As seen in Figure 11a, the thick portion of the shell was utilized to produce five Type III dogbone specimens where the tensile strength was measured perpendicular to the direction of deposition. The flat panel shown in Figure 11b was printed for five dogbone specimens where the tensile strength was measured in the direction of deposition. As seen in Figure 5a, supports were added to make the print geometry viable. The flat panels, with a 16 mm thickness (Figure 5b), were machined to 10 mm in uniform thickness, to meet ASTM D638-14 Type III dogbone requirements [51] and limit the impact of bead-geometry-induced stress concentrations. The dogbones were cut from the samples in the direction of the printed lines. The vertical print was waterjet cut into 10 mm strips, and each one was then cut into the desired dogbone geometry. For each of the five dogbone specimens in each direction relative to the deposition for mechanical testing, tensile testing was conducted in accordance with Type III dogbone ASTM D638-14 [51] to produce a tensile strength correlated with an EM and fiber alignment to determine the interrelationship behavior. Due to printer-specific limitations, not all EMs were tested in both directions, with the EM of 1.2 only being tested in the direction of deposition. The tensile testing provided variation in part properties for the given extrusion multiplier parameter set. Figure A1 shows the orientations of the dogbones that were desired. Using an Instron Electromechanical Test Frame (68FM-100), the dogbones were tested until failure to determine the ultimate tensile strength. For isotropy comparison purposes, five Type V dogbones were injection-molded to evaluate the tensile testing results in an assumed isotropic sample space and tested in accordance with the same ASTM standard, D638-14, on an Instron ElectroPuls. While the difference in varying geometries may produce a difference in performance, it was deemed negligible in favor of minimizing the material usage.

## 3. Results and Discussion

### 3.1. Fiber Alignment Process to Microstructure Relationship Development

#### 3.1.1. X-ray Microscopy

A sample from each of the EM prints was scanned in the XRM to determine the fiber alignment. Due to noise in the sample, there were single voxels assigned as a pore that were a polymer or fiber. To correct for this, a volume filter was used to ignore objects that were less than 9 voxels. From the deep learning model, the measured volume composition of each sample is reported in Table 1.

As reported in Table 1, the small standard deviation for the compositions of the samples provides confidence in the segmentation method, because it is assumed that the physical samples had a similar composition, with some variation due to the sample distribution and variability in the scan location relative to layer structures. The manufacturer of the pellet feedstock reported a carbon fiber percentage of 10%; it was not disclosed if this is a mass or volume percentage. The manufacturer-reported fiber percentage is within 1% and 1 standard deviation of the measured fiber volume percentage. Using the XRM processing script (Appendix D), the fiber alignment for each EM is reported in Table 2.

**Figure 12:**
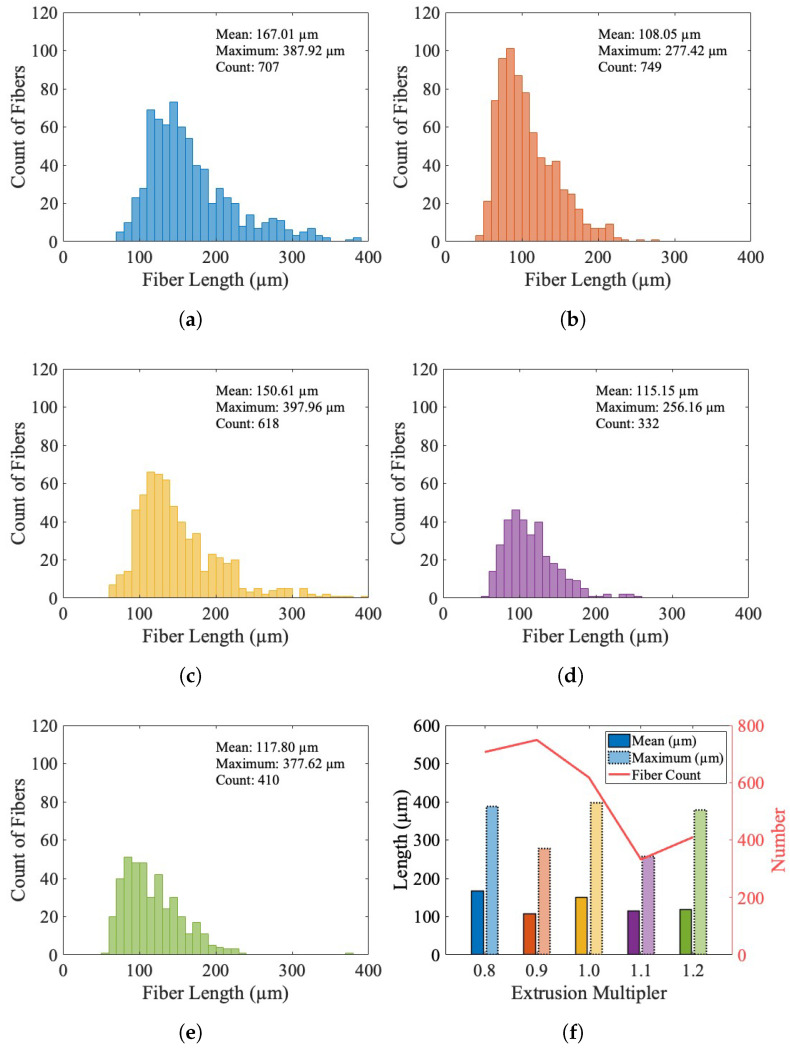
Histograms of X-ray microscopy fiber lengths for each extrusion multiplier. (**a**) 0.8 extrusion multiplier. (**b**) 0.9 extrusion multiplier. (**c**) 1.0 extrusion multiplier. (**d**) 1.1 extrusion multiplier. (**e**) 1.2 extrusion multiplier. (**f**) Summary of distributions showing the mean and maximum fiber length in each scan, where the various colors are for each scan.

Each value reported is an average for all objects in each scan that pass the volume thresholds. The object data were exported for an evaluation of the assumption of the fiber length in the discrete image analysis method and the uniformity of the segmentation across the EMs. Figure 12 shows the fiber length distributions for all extrusion multipliers. It is notable that all EMs show a bias towards shorter fibers, with an average fiber length across all EMs of 131.72 ± 25.64 μm.

There is no significant trend behavior for the mean or maximum fiber length for the different EMs, as shown in Figure 12f. While the count of recognized objects is largely variable, the distributions of length are similar for the EMs, as shown in Figure 12a–e, providing confidence that the segmentation method uniformly segmented the different scans. This is because if it was improperly over-segmenting fibers for a specific EM, the distribution would see a stronger bias to a shorter length, and if it was improperly under-segmenting fibers for a specific EM and reporting several fibers as a single object, the bias would shift to a longer length.

#### 3.1.2. Image Analysis

The reported values in Table 3 are the averages of five slices for each EM. The slices were taken from a single plane and dispersed throughout the thickness of the sample, as shown in Figure 7b. The standard deviation reported is the standard deviation in orientation for all slices relative to the mean reported value.

### 3.2. Validation of 2D Discrete Image Analysis—Microstructure Technique Comparison

**Figure 13:**
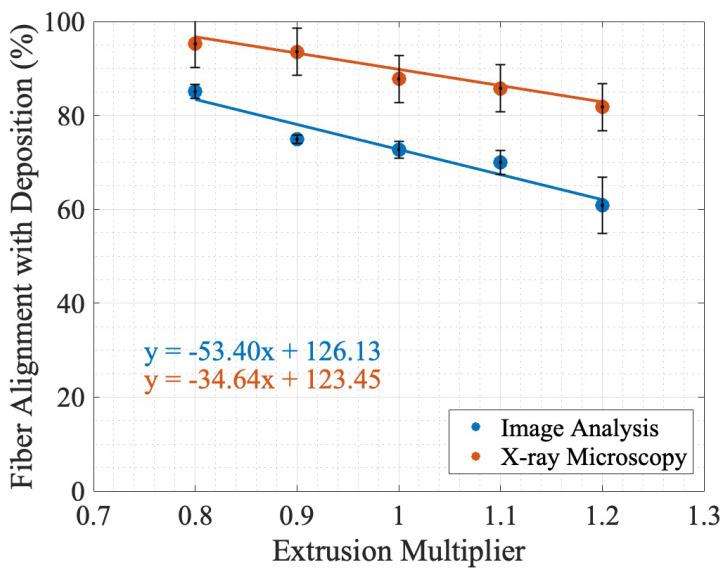
Comparison of fiber alignment using simple linear regression in the direction of deposition from image analysis and XRM.

The uncertainty in the measurements of the fiber alignment in Figure 13 for the image analysis is the standard deviation in the object orientation, as reported in Table 3. For XRM uncertainty, a 5% uncertainty bound was assumed from both the volume and size thresholding. It was expected that the values would not agree for the two methods due to the varying thresholding techniques for image analysis and XRM analysis. Furthermore, the shift between the discrete image analysis and X-ray microscopy lines of best fit, where XRM reports higher values for every extrusion multiplier, could be due to the inability of a 2D approximation using a length assumption to correct for all fibers in an image with a baseline orientation shift and no fibers parallel to the image plane.

As seen in Figure 13, there is an inverse relationship between the fiber alignment in the direction of deposition as a function of the EM, with an $R^{2}=0.97$ and $R^{2}=0.92$ for the lines of best fit from XRM and image analysis, respectively. This relationship was hypothesized in Figure 2 and verified by the negative slope of the linear regression for both XRM and image analysis. As shown in Figure 13, the two methods do not have agreement in value or slope, as shown in the discrepancy between −34.64 $\frac{\%}{EM}$ and −53.40 $\frac{\%}{EM}$ for X-ray microscopy and image analysis, respectively, and the lack of overlapping uncertainty bounds. While this simple linear regression is the line of best fit for the given recorded values, it did not account for the variation in possible values due to uncertainty.

**Figure 14:**
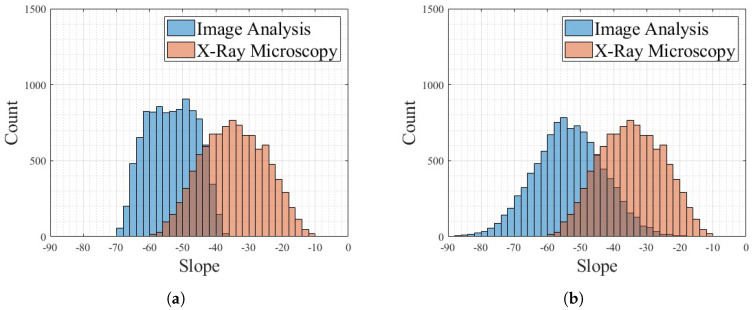
Monte Carlo simulation of slopes for validation, where both random (**a**) and normal (**b**) uncertainty profiles were simulated for the XRM results. Given the image analysis uncertainty is from the standard deviation, for both simulations it followed a normal distribution uncertainty profile.

The mean slopes from the Monte Carlo simulation for select permutation counts are reported in Table 4. As reported in Table 4, there is overlap in every tested permutation count. Figure 14 shows the histogram of slope values for 10,000 permutations, with Figure 14a showing the distribution of slopes when assuming both the image analysis and XRM methods have a random distribution of values in their respective uncertainty bounds, and Figure 14b shows image analysis where there is a normal distribution within the uncertainty bounds and XRM has a random distribution. Figure 14b shows image analysis as a normal distribution because it is a standard deviation-based uncertainty, whereas the XRM is based on the resolution of the instrument and error in segmentation and is assumed to be a random distribution [52]. As seen in Figure 14, all permutations show a negative slope for both methods and probability distributions. The distribution of slopes for image analysis does not follow a random distribution, unlike the XRM slope distribution in Figure 14a. This is caused by the non-uniform uncertainty bounds for each data point in the image analysis, whereas the XRM analysis assumed uniform uncertainty bounds for all data points. The overlap between the slope distribution of the image analysis and the XRM analysis shows that, given the uncertainty bounds and chosen probability distributions and the fact that a given data point falls within its uncertainty bounds in either a random or a normal probability distribution, there is agreement between the two methods of determining fiber alignment. Future efforts to decrease the uncertainty bounds would decrease the width of the distribution of slopes and provide conclusive evidence on the accuracy of a 2D image-based analysis for 3D fiber representation and analysis.

### 3.3. Mechanical Testing—Process to Property Relationship Development

**Figure 15:**
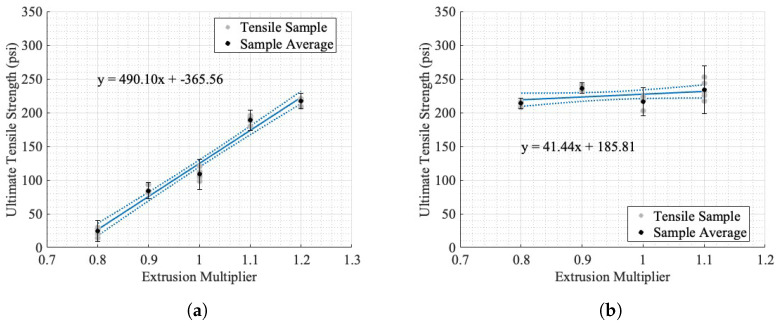
Extrusion multiplier vs. ultimate tensile strength, with 95% confidence intervals and sample averages: (**a**) perpendicular to direction of deposition; (**b**) parallel to direction of deposition. The solid lines denote the line of best fit and the dashed lines show the width of the 95% confidence bounds.

The results of the tensile testing in the two orientations are shown in Figure 15, where the line of best fit is shown and incorporates all the sample values, not the mean value shown.

The uncertainty bounds in Figure 15 are the instrumentation uncertainty as calculated in Appendix B.1 in addition to the uncertainty due to the variation in the sample ultimate tensile strength. The line of best fit is shown, as well as 95% confidence bounds for both orientations of dogbones. The evlaution criteria for the lines of best fit are reported in Table 5.

The relationship between the EM and the ultimate tensile strength perpendicular to the direction of deposition (Figure 15a) is approximately linear, with an $R^{2}$ of 0.97. While these samples produce a linear relationship, it is likely that there is a non-linear region above or below the tested EM. As seen in Figure 6, the EM tests ranged from an irregular bead placement from under-extrusion to solid-like parts. The x-intercept of this linear fit is an EM of 0.75, corresponding to a theoretical 0 psi ultimate tensile strength in the build direction. While this was not tested, the initial testing for determining a valid EM (Figure 6) showed that an EM of 0.7 could not withstand the required machining process and fractured under cutting and polishing. The direction of deposition shows an irregular relationship between the ultimate tensile strength and EM, with a linear relationship plotted in Figure 15b to highlight the lack of linearity in comparison with Figure 15a. Furthermore, the $R^{2}$ value was 0.13. The two orientations of dogbones showed relatively similar maximum ultimate tensile strengths, with perpendicular to deposition being 217.16 ± 0.07 psi at an EM of 1.2 and parallel to the direction of deposition being 236.4 ± 0.08 psi at 0.9 EM.

#### Tensile Isotropy Analysis

**Figure 16:**
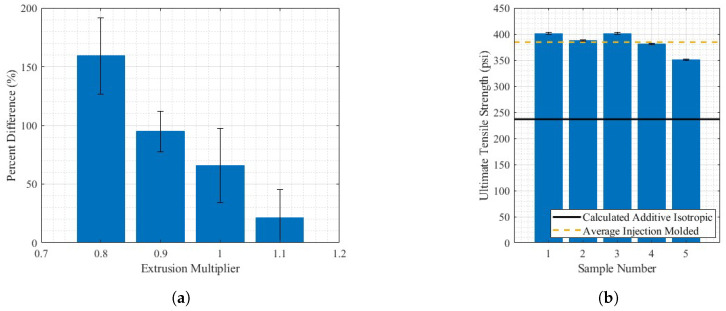
(**a**) Isotropy ultimate tensile strength analysis through percent difference between direction of deposition and perpendicular to deposition and (**b**) injection-molded tensile testing results in comparison with estimated isotropic additive part.

The injection-molded ultimate tensile strength was 384.49 ± 33.55 psi, where the uncertainty is the maximum distance from the mean to any sample result. As seen in Figure 15, the difference in tensile strength for the two orientations changes as the EM increases. At a low EM, the resultant part is extremely anisotropic, as shown by the differencial in ultimate tensile strength between the two orientations at an EM of 0.8. As the EM increases, the ultimate tensile strength anisotropy decreases. The percent differences between the average ultimate tensile strengths for all five samples at each orientation are reported in Figure 16a.

In Figure 16a, the decrease in the percent difference is likely reflective of the increase in the bonding and volume of material in the perpendicular to deposition orientation. This is supported by Ghorbani et al., who studied the relationship between layer interfaces and mechanical orthotropy in fused filament printing [53]. This, however, is not conclusive because this study was completed on small-scale printers. The theoretical ultimate tensile strength of a completely isotropic part, with a percent difference in UTS of 0%, is the intersection point of the two lines of best fit in Figure 15 at an EM of 1.23 and an ultimate tensile strength of 236.78 ± 43 psi, where uncertainty was determined using the maximum uncertainty for any point reported in Figure 15 for both lines of best fit to determine the overlapping regions. It is noteworthy that this value extrapolates outside the tested samples and assumes a constant linear trend as the EM increases. The results of the injection-molded tensile testing are reported in Figure 16b, for comparison with an estimated isotropic additive tensile sample. It is important to note that the sample size of the injection-molded samples, Type V, was dimensionally smaller than the Type III tested for the two orientations of printed samples. While both testing methods are normalized based on their cross-sectional area, it may influence the comparison of results. The difference in estimated additive isotropic and average injection-molded tensile strengths, as shown in Figure 16b, shows that the material is capable of a higher ultimate tensile strength than is produced from an estimated isotropic printing process. This evaluation method assumes that the two orientations of dogbones have similar mechanical properties in all directions, due to having the same print conditions except the geometry. The prints with higher EMs tested, from visual inspection in Figure 6d,e are cohesive and not subject to large print-specific features, such as voids. This shows that the difference in ultimate tensile strength for printed samples and injection-molded samples is not only dependent on the mesoscale density of the material and is likely resultant of the time-based deposition process in printing as opposed to the near-immediate injection molding and the dispersion of fibers through the entirety of an injection-molded sample, whereas in AM, short fibers cannot bridge across layers.

### 3.4. Fiber Alignment and Tensile Strength—Structure to Property Relationship Development

The relationship between the fiber alignment and ultimate tensile strength is reported in Table 6.

**Figure 17:**
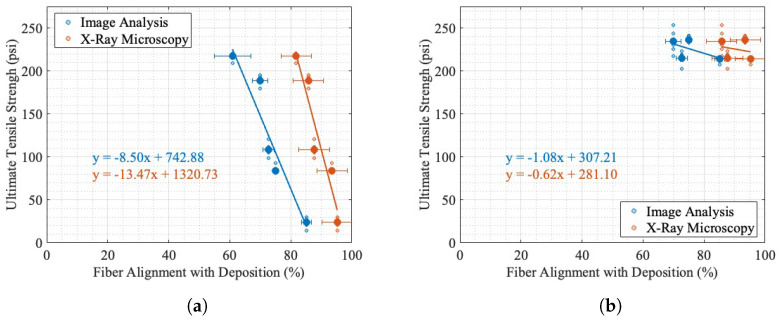
Ultimate tensile strength vs. fiber alignment in the direction of deposition, showing simple linear regression and sample averages: (**a**) perpendicular to direction of deposition; (**b**) parallel to direction of deposition.

As predicted, as the fiber alignment increases in the direction of deposition, the ultimate tensile strength decreases perpendicular to the direction of deposition, as seen in Figure 17a. This was expected based on a visual inspection of the previous prints shown in Figure 6, where, as the EM increased, there was a visible increase in the bead contact area from a layer to subsequent layers. As reported in the literature [24,25,26,27,28,29,30,32,33,34,35], fibers aligned in the direction of testing can produce higher tensile strengths in that direction. Huang et al. found in Ultra-High-Performance Concrete that the alignment of short fibers could increase the tensile strength in that direction between 30 and 90%  [24]. Fu et al. describes the strength of a short fiber–polymer composite as being derived from the fiber length and orientation and uses these critical values to model and predict the tensile strength [25]. Banaker et al. explored epoxy resin composites, determining there are optimal fiber orientations for laminate mechanical performance and that the fiber orientation had a significant impact on the tensile strength [26]. Similarly, Punyamurthy et al. found that in composites, an increase in the fiber orientation in the direction of testing showed both an increase in the tensile strength and Young’s modulus in abaca fiber and E-glass fiber-reinforced epoxy resins [27]. However, laminate epoxy composites with continuous fibers have different properties than SFTs. Wang et al. found that with short glass fiber polyamide-6, the tensile strength increased by a factor of 2 between a 0-degree and 90-degree fiber alignment with the test direction [28] . Mortazavian et al. studied short glass fiber polybutylene terephthalate and short glass fiber polyamaide-6 at various temperatures and experimentally showed that for nearly all the temperatures, a higher fiber orientation in the direction of testing produced a higher tensile strength for both materials [29]. Kim et al. found polymer composites had increased tensile strength with a higher short fiber volume content and an alignment of the fiber orientation with the direction of loading [30]. The alignment of fibers in the direction of deposition was experimentally shown in Badini et al. for a short fiber fused filament fabrication printing process [31], where it was compared to a selective laser sintering additive, which was found to produce a more chaotic fiber orientation distribution. In additive applications, most fiber orientation and mechanical strength correlation is based on the assumption that fibers align in the direction of deposition, such as in Ivey et al. [32], Spoerk et al. [33], Russell et al. [34], and Ferreira et al. [35], which found a correlation between the deposition direction and thus the assumed fiber alignment in short fiber thermoplastics and an increased mechanical performance in stiffness and tensile strength. This relationship was not observed for the EMs tested, as shown in Figure 17b. In summary, there is a large amount of research in both composites and AM that support the hypothesis that as both short and long continuous fibers align in the direction of tensile testing, the ultimate tensile strength increases. However, as seen in Figure 17b, as the fiber alignment with the direction of deposition increased, the ultimate tensile strength had a weakly correlated negative relationship with the fiber alignment for both image analysis and XRM.

The disagreement with the literature that the fiber alignment in the direction of testing increases the ultimate tensile strength, as seen in Figure 17b, is likely reflective of the difference in material density as the EM varies. A visualization of the part density as a function of the EM can be seen in Figure 6. While the fiber alignment decreases in the direction of deposition as the EM increases, the material meso-density from the structure (i.e., bead geometry and voids) increases the dogbone strength, possibly causing the lack of variation in ultimate tensile strength in Figure 15b. This relationship impacts the results of Figure 17b. The decrease in ultimate tensile strength as the fiber alignment increases, as seen in Figure 17a, is likely associated with the decrease in layer adhesion and bead bonding as the bead cross-sectional area decreases as the EM decreases, as seen in Figure 6 and reported in Ghorbani et al., which found that an increase in the EM decreases mesoscale print-induced features, like the void space seen in Figure 6a [53].

### 3.5. Density Image Analysis to Ultimate Tensile Strength—Micro- and Meso-Structure to Property

The results of the mesoscale density image analysis for the EM are reported in Table 7.

Previous work has found a decrease in tensile strength with an increase in voids in composites [54], particularly in additive manufacturing [55,56], which showed a decrease in mechanical performance as large print-induced structures like voids increased. Zhu et al. [54] found that in carbon/epoxy laminate composites, as the void content increased, the mechanical strength decreased. For the additive manufacturing of a silicon cured elastomer, Plott et al. [56] found that the void size has a significant impact on the UTS, and, as the size of the voids varied, the failure mechanism changed. Tronvoll et al. [55] found in fused filament small-scale AM that the process-induced voids between beads and layers, as seen in Figure 6a, are a large contributor to the tensile anisotropy associated with AM parts. However, the relationship of mesoscale voids to the UTS in LFAM has still not been established.

**Figure 18:**
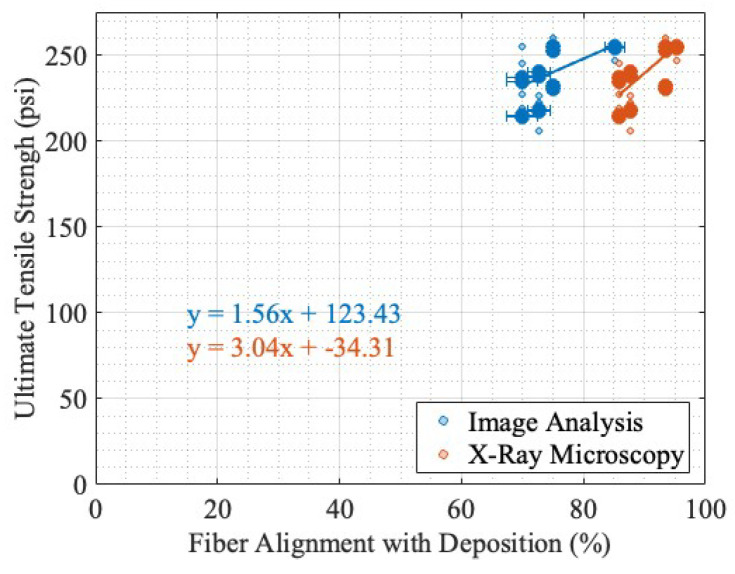
Parallel to direction of deposition with modified cross-sectional area for material composition.

The adjustment of the load-bearing percent for the direction of deposition produces a positive slope for both the image analysis and XRM-reported fiber alignment, as shown in Figure 18, an inverse of the linear regression in Figure 17b, and shows that the mesoscale structure of the dogbones (i.e., voids and beads) and the structure of the additive parts may be the cause of the unexpected behavior in comparison with the literature. The findings show that the cause of the discrepancy may be a meso-structure-induced decrease in ultimate tensile strength, not a microstructure dependent on the fiber alignment. The $R^{2}$ values of the adjusted cross-sectional area fiber alignment to the ultimate tensile strength are 0.29 and 0.52 for image analysis and XRM, respectively. Both $R^{2}$ values increased with the adjustment for the load-bearing area.

The agreement with the literature shows a need for micro-to-meso-translations for property evaluation in LFAM. While injection molding produces a more uniform bulk part, the induced meso-structure of voids in LFAM parts creates meso-structure dominant properties that relate to microstructure variation. A possible test method to limit this would be to print singular beads for analysis or adjust the tool path for mesoscale density, which is visualized in Figure 10c. This approach could be performed through the adjustment of the slicer bead width and maintaining a constant EM, and the various EMs would produce dogbones with different counts of beads for the same geometry. The interrelationships between the microstructure, being fiber alignment within a bead, and meso-structure, being the variable voids as a function of the EM, and their influence on the measured ultimate tensile strength show that property evaluation in LFAM may not be able to be treated the same as traditional processing methods, like injection molding.

## 4. Conclusions

This work developed a series of process–structure–property relationships for large-format additive manufacturing. The process-dependent properties of LFAM poly(lactic acid) with short carbon fiber are shown through the variation in ultimate tensile strength for the two orientations of dogbones relative to the print. The comparison of the estimated additive isotropic tensile strength for injection molding shows the possibility that the time-based nature of deposition in additive manufacturing introduces tensile weakness that is not correlated with visible print-induced features, like mesoscale voids. Linear fits were produced for various related variables, like the EM and fiber alignment, and the respective $R^{2}$ values are reported as metrics of correlation. Some findings of this paper are listed below:

- The use of density-based segmentation methods alone for XRM is not applicable to this composite polymer system, and a deep learning model needed to be applied for proper segmentation.
- For all the EMs tested, both XRM and image analysis methods showed a decrease in fiber alignment in the direction of deposition as the EM increases, as predicted.
- For the utilized thresholding techniques, the image analysis method is validated for predicting linear trend behavior by the XRM analysis via a Monte Carlo simulation to account for uncertainty bounds. Further comparison and an increased number of data points could produce a thinner distribution of possible slopes and further validate the image analysis method.
- For all the EMs testeds, as the EM increased, the tensile anisotropy of the dogbones decreased.
- The injection-molded samples had a higher ultimate tensile strength than the estimated isotropic additively manufactured ultimate tensile strength.
- The fiber alignment did not have the expected relationship reported in the literature with the ultimate tensile strength in the direction of deposition. This is possibly due to the relationship between the EM and mesoscale printing-induced features, such as the void content.

This work presents a series of linear fits for carbon fiber PLA to show trend behavior of varying the extrusion rate in LFAM on the microstructure and meso-structure and their resultant impact on the mechanical tensile performance. The process–structure–property relationship development in this work is a novel approach to LFAM and will help guide process control for predictable performance. Future work in the process–structure–property relationship development for LFAM is required to better understand other material properties, like the compression and flexural strength, material systems, induced structures, and production of parts with a predictable and desired performance. The application of this work is the ability to customize not only the geometry of an LFAM part but also the mechanical performance by adjusting the processing for specific use cases.

## Figures and Tables

**Table 1:** Composition by volume of XRM samples.

Extrusion Multiplier	Polymer (%)	Pore (%)	Fiber (%)
0.8	87.20	5.42	7.37
0.9	82.84	4.64	12.52
1.0	85.56	5.42	9.03
1.1	83.67	5.71	10.62
1.2	84.44	5.63	9.65
Average	84.74	5.36	9.84
Standard Deviation	1.53	0.38	1.71

**Table 2:** Average fiber alignment from XRM.

Extrusion Multiplier	Direction of Deposition (% ± 5%)	Perpendicular to Deposition (% ± 5%)
0.8	95.21	4.79
0.9	93.54	6.46
1.0	87.76	12.30
1.1	85.76	14.24
1.2	81.78	18.22

**Table 3:** Average fiber alignment from image analysis.

Extrusion Multiplier	Direction of Deposition (%)	Perpendicular to Deposition (%)	Standard Deviation (%)
0.8	85.11	14.89	1.53
0.9	74.95	25.05	0.91
1.0	72.70	27.30	1.80
1.1	70.01	29.99	2.52
1.2	60.88	39.12	5.96

**Table 4:** Summary of Monte carlo convergence test for 2D code validation.

Permutations	Image Analysis Mean Slope ($\frac{\%}{\mathrm{EM}}$)	X-ray Microscopy Mean Slope ($\frac{\%}{\mathrm{EM}}$)	Overlap
10	−53.35	−34.54	Yes
100	−53.90	−32.81	Yes
1000	−53.58	−34.85	Yes
10,000	−53.51	−34.70	Yes

**Table 5:** Line of best fit results for tensile testing for both orientations.

Orientation	Slope ($\frac{\mathrm{psi}}{\mathrm{EM}}$)	$R^{2}$	Standard Error
Perpendicular to deposition	490.10	0.97	18.09
Parallel to deposition	41.44	0.13	25.01

**Table 6:** Line of best fit results for ultimate tensile strength and fiber alignment relationships.

Analysis	Orientation	Slope ($\frac{\mathrm{psi}}{\%}$)	$R^{2}$	RMSE
Image analysis linear regression	Perpendicular	−8.50	0.89	24
	to deposition			
	Parallel to	−1.08	0.24	11.7
	to deposition			
XRM linear regression	Perpendicular	−13.47	0.90	22.8
	to deposition			
	Parallel to	−0.62	0.04	13.2
	to deposition			

**Table 7:** Estimated material percent in tensile sample cross-section.

Extrusion Multiplier	Load-Bearing Cross-Section (%)
0.8	84.2
0.9	92.6
1.0	98.5
1.1	99.3
1.2	99.8

## Data Availability

Data are contained within the article.

## Abbreviations

    The following abbreviations are used in this manuscript:

MEX	Material extrusion
UTS	Ultimate tensile strength
LFAM	Large-format additive manufacturing
SFT	Short fiber thermoplastics
SF	Short fibers
XRM	X-ray microscopy
T_g_	Glass transition temperature
EM	Extrusion multiplier
PLA	Polylactic acid
T_melt_	Nozzle temperature, last heat band temperature before extrusion
CAD	Computer-aided design
ROI	Region of interest

## Appendix B. Uncertainty Estimations

### Appendix B.1. Ultimate Tensile Strength

(A1) $$UTS=\frac{L}{cal^{2}}$$

where the *UTS* is the ultimate tensile strength, *L* is the load, and *cal* is the caliper-measured inches for the cross-sectional area.

(A2) $$\delta UTS=\pm \sqrt{\left({\frac{\delta UTS}{\delta L}}^{2}\right)*\delta L+\left({\frac{\delta UTS}{\delta cal}}^{2}\right)*\delta cal}$$

where δ*UTS* is the uncertainty in the ultimate tensile strength in psi, $\frac{lbs}{in^{2}}$ δ; *L* is the uncertainty in load measurements, reported by the manufacturer as 0.5% of the measured load; δ*cal* is the uncertainty in caliper measurement, reported by the manufacturer as ±0.001 inch; $\frac{\delta UTS}{\delta L}$ is the uncertainty and partial derivative of the UTS with respect to the load; and $\frac{\delta UTS}{\delta cal}$ is the uncertainty and partial derivative of the UTS with respect to caliper measurements.

(A3) $$\frac{\delta UTS}{\delta L}=1$$

(A4) $$\frac{\delta UTS}{\delta cal}=\frac{-2}{cal^{3}}$$

(A5) $$\delta UTS=\pm \sqrt{{\left(0.005\right)}^{2}*L+{\left(\frac{-2}{cal^{3}}\right)}^{2}*0.001}$$

## Appendix C. Percent Difference Calculations

**Table A1:** Percent difference in tensile strengths.

EM	Perpendicular to Deposition (psi)	Parallel to Deposition (psi)	Percent Difference (%)	Uncertainty (%)
0.8	24.32	213.69	159.13	32.63
0.9	84.22	236.40	94.93	17.10
1.0	108.37	214.67	65.81	31.35
1.1	188.63	234.15	21.54	23.80
1.2 *	217.16	235.54	8.12	14.40

* Where the uncertainty in EM 1.2 in the direction of deposition is assumed to be equal to the maximum uncertainty in the direction perpendicular to deposition. The ultimate tensile strength perpendicular to deposition for a 1.2 EM is experimental, and the parallel to deposition ultimate tensile strength was estimated by the line of best fit reported in Figure 15b due to a lack of experimental data for this EM and orientation.

## Appendix D. Portions of XRM Processing Script: MATLAB

% Variable adjustment is required based on

% File specific details

multiroi=table2array(readtable("[Insert File Name]"));

% Volume - used for threshholding a group of

% connected fibers AND static

% min and max xyz are used to determine R

% in spherical coordinates

% phi and theta are used with R

% to determine cartesian values

r=sqrt((xmax-xmin).^2+(ymax-ymin).^2+(zmax-zmin).^2);

% This is an estimated R that neglects the thickness of the

% fiber as significant

for i = 1:1:length(multiroi)

  if multiroi(i,4) < 3e-5 \&\& multiroi(i,4) > 6e-6

     fibers(i,:)=multiroi(i,:);

  else

     continue

  end

end

  x=r*sin(fibers(:,5)).*cos(fibers(:,6));

  y=r*sin(fibers(:,5)).*sin(fibers(:,6));

  z=r*cos(fibers(:,5));

% Normalized these values and reported components

## Appendix E. Monte Carlo Simulation Script

 Nperm=10000; % 10,000 Monte Carlo simulation permutations

 % For Image Analysis Random Distribution

  for i=1:1:Nperm

    indir2D=indir+[1.53-(randperm(306,1)/100) 0.91-(randperm(182,1)/100),

    ... ,1.8-(randperm(360,1)/100) 2.52-(randperm(504,1)/100), ...

    ,... (5.96-randperm(1192,1)/100)]; % standard deviation

    mdlran=fitlm(EM,indir2D);

    modeleqn=table2array(mdlran.Coefficients(:,1));

    storeallmodeleqn(i,:)=modeleqn;

    slopes(i,:)=mdlran.Coefficients(2,:);

  end

 slope2mean=mean(slopes.Estimate)

 slope2std=std(slopes.Estimate);

 % For XRM Random distribution

  for i=1:1:Nperm

    indir3D=indir3+5-randperm(10,5); % based on +/- 5% assumption

    mdlran3=fitlm(EM,indir3D);

    modeleqn3=table2array(mdlran3.Coefficients(:,1));

    storeallmodeleqn3(i,:)=modeleqn3;

    slopes3(i,:)=mdlran3.Coefficients(2,:);

  end

 slope3mean=mean(slopes3.Estimate)

 slope3std=std(slopes3.Estimate);

% For Image Analysis Normal Distribution

for i=1:1:Nperm

   out=error2D.*randn+indir; %randomly selects values given a ...

   % normal distribution of

   % uncertainty bounds

   mdlran=fitlm(EM,out);

   modeleqn=table2array(mdlran.Coefficients(:,1));

   storeallmodeleqn(i,:)=modeleqn;

   slopesNormal(i,:)=mdlran.Coefficients(2,:);

end

## Appendix F. Image Processing for Load Bearing Cross-Section

% Area Calculation for Tensile Normalization

image=imread(’1.2ForMATLAB\.jpg’);

numpixel=size(image,1)*size(image,2);

pic1=graythresh(image);

   bw=im2bw(image,pic1);

   figure(2)

   imshow(bw)

bw=bwareaopen(bw,[Insert Desired pixel connectivity]);

  %removes all connected components that have fewer than X pixels

object=bwconncomp(bw);

  % Finds connected components in image to give count of number of

  % objects and other properties

objdat=regionprops(object,’Area’);

  %This makes a data matrix that takes the objects created above for the

  %obj and stores characteristic properties as listed

area=[objdat.Area];

voidcontent=sum(area,2);

materialpercent=(numpixel-sum(area,2))/numpixel
